# A Refined Finite Element Formulation for the Microstructure-Dependent Analysis of Two-Dimensional (2D) Lattice Materials

**DOI:** 10.3390/ma6010001

**Published:** 2012-12-20

**Authors:** Geminiano Mancusi, Luciano Feo

**Affiliations:** Department of Civil Engineering, University of Salerno, Fisciano 84084, Italy; E-Mail: l.feo@unisa.it

**Keywords:** lattice materials, band gaps, couple stress theory, finite element analysis

## Abstract

A finite element approximation is proposed for the dynamic analysis of two-dimensional (2D) lattice materials. The unit cell is modeled by means of a defined number of shear deformable micro-beams. The main innovative feature concerns the presence of a microstructure-dependent scale length, which allows the consideration of the so called size-effect that can be highly relevant, due to the characteristics of the lattice at the local scale. Some numerical results show the influence of the microstructure parameter on the dynamic behavior of two-dimensional lattice materials.

## 1. Introduction

At the local scale, many periodic materials can be seen as made of interconnected beams. Such materials, also called lattice materials [1,2], have received a wide interest in the scientific and industrial community over the last years, mainly due to their high strength to weight and stiffness to weight ratios, which, of course, are an important advantage from a static point of view. Lattice materials, in fact, can be designed to be stretching-dominated, thus providing higher structural performances. Many scientific papers dealing with the characterization of their effective elastic properties are available in the literature [3,4,5,6,7]. In comparison, only a few papers focus on their dynamic behavior, which is of an identical practical interest in the engineering field. Innovative applications are, in fact, related to many aspects concerning the elastic wave propagation mechanism within them. A small number of recently published works [8,9,10,11,12] has pioneered the study of the wave propagation phenomena in two-dimensional (2D) periodic lattice materials, with the main scope of detecting the existence of phononic band gaps,* i.e.* frequency ranges, within which the propagation of elastic/acoustic waves is prevented regardless of the incident wave direction. These studies look at a new generation of devices for energy absorption, noise and vibration control. Moreover, very interesting results have also been established within the field of civil engineering, where a new seismic mitigation strategy has been proposed that exhibits a close analogy with the idea of a lattice material [13].

In the context of lattice materials, the periodic topology plays a relevant role, considering the strict relation with the bending mechanism, which can be significantly dominant at the micro scale, where both the displacements and rotations can be similarly important [14]. This, in addition to experimental evidence reported by many authors [15,16,17,18,19], suggests an overcoming of the classical continuum theory, which assumes only displacements as kinematic quantities.

According to the classical continuum theory, stress-transfer locally occurs only through a force vector per unit area. As a consequence, stresses and strains can be represented by symmetric second-order real tensors. Nevertheless, for the accuracy of any prediction given via the classical theory, the condition that changes in stresses and strains (with wavelength *λ*) can be considered as uniform at the local scale is pivotal: D > *λ* ≫ *l*, with D denoting the global (structural) size, while *l* is a characteristic length representative of the microstructure. On the contrary, when dealing with a number of situations [20,21,22,23,24] (thin films, adhesive interfaces, notches, crack tips, localized deformations, boundary layers), it can be observed that the scale lengths of the problem agree with a new different condition (D > *λ* > *l*), rather than the previous one (D > *λ* ≫ *l*). More precisely, the lower the ratio *λ*/*l* > 1.0, the greater the importance of considering non-uniform stresses and strains at the local scale.

Unfortunately, due to the lack of an internal characteristic length, classical models are unable to capture the microstructure-dependent size-effect and, therefore, need to be extended by using higher order, non-local continuum theories.

Many continuum theories have been introduced over time in order to account for the size-effect [25,26,27,28,29,30,31,32]. Recently, non-local theories for the Bernoulli–Euler [33] and Timoshenko [34] beams have been proposed according to a modified couple stress theory [35] by using the non-local constitutive equation introduced in [30].

In line with these last developments, the present work proposes the analysis of the microstructure-dependent size-effect on the dynamic behavior of 2D lattice materials.

Due to the very important conclusions outlined in [33,34], this influence is expected to be relevant also on the local resonance phenomena, which determine the existence of frequency band gaps within the low frequency region.

To this scope, a refined 1D finite element approximation, accounting for a micro-scale length parameter, as well as the shear deformability, has been proposed.

## 2. Frequency Gaps

Due to the complexity of the equations governing the wave propagation within a periodic material, there is no general rule that can be used to predict whether phononic band gaps exist before a full analysis has been performed.

The existence of phononic band gaps is commonly related to the destructive interference occurring in the multiple scattering and reflection of elastic waves, which propagate throughout a periodic material. It is well known that the position and width of the phononic band gaps depend on the lattice topology, as well as the elastic moduli of the material and the mass density. Nevertheless, the influence of the micro-scale constitutive parameters has yet to be analyzed in detail.

Recent experimental and numerical studies [36,37,38], however, show the existence of phononic band gaps in frequency ranges lower than those evaluable from the above cited destructive interference theory, revealing the link between these low frequency band gaps and the local resonance phenomena of the inner microstructures. Moreover, in a recent work [39], a modified 2D lattice material with square topology, obtained by connecting auxiliary cantilever beams to the primary micro-structure, was investigated. The frequency band diagrams, numerically identified, show as a result the relevant role played by the physical mechanism of local resonance.

Based on the previous considerations, it is the opinion of the authors that the study of the dynamic response of 2D lattice materials should require a more refined continuum approach, which accounts for the stiffness modification related to the size-effect. To this aim, a micro-scale length parameter has been proposed according to the hypotheses of the modified couple stress theory developed in [35].

## 3. Free Waves Propagation in 2D Periodic Lattice Materials

**Figure 1:**
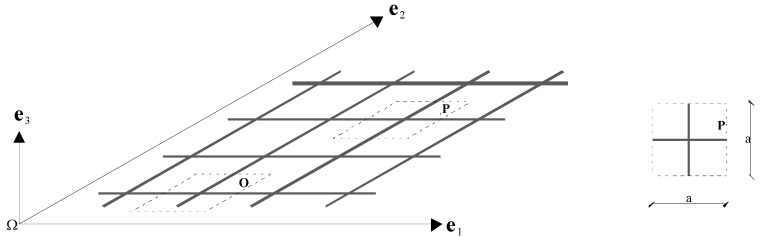
Example of two-dimensional (2D) square lattice material.

The study of the wave propagation within a periodic material generally involves the Bloch’s theorem, which allows one to reduce the analysis of an infinite lattice to that of a single unit cell. An example of a simple two-dimensional square lattice material and the corresponding unit cell is depicted in Figure 1, where **e**_1_ and **e**_2_ are the unit vectors along the directions of spatial periodicity. The square topology implies in this case that **e**_1_ and **e**_2_ are normal to each other. 

In the example above (Figure 1), the microstructure is composed of interconnected beams arranged along the directions **e**_1_ and **e**_2_ only. In general, however, more complex microstructures can be investigated. For instance, in [39], a modified 2D lattice material has been considered, which exhibits a more elaborated unit cell.

According to the Bloch’s theorem, if a plane elastic wave with angular frequency *ω* propagates in the lattice material (Figure 1), the displacement of an arbitrary point **P** is given by:

(1) $$u\left(r\right)=u_{k}\left(r\right)\exp\left(-i\omega t+k\cdot r\right)$$

In Equation (1), the symbol **r** indicates the position of the point P, **k** denotes the Bloch wave vector and *u_k_* (*r*) is the amplitude, which is characterized by the same spatial periodicity as the point lattice. As a consequence, it is possible to express the position of any point **P** as a function of the position of the corresponding point **O**, which is located in a reference unit cell. Once the reference cell has been selected (see Figure 1), it results: **r** = **r**_0_ + *n*_1_**e**_1_ + *n*_2_**e**_2_, where (*n*_1_, *n*_2_) is an integer pair and **r**_0_ is the position vector of **O**. It is then possible to update Equation (1) as follows:

(2) $$u\left(r\right)=u\left(r_{0}\right)\exp\left(n_{1}k\cdot e_{1}+n_{2}k\cdot e_{2}\right)$$

In brief, changes in wave amplitude from a generic cell to the adjacent one do not depend on the cell location within the point lattice. By virtue of this, it is possible to restrict the study of an infinite lattice system to the analysis of a unit cell.

It is easy to verify that the reciprocal unit vectors ${\hat{e}}_{1}$ and ${\hat{e}}_{2}$ assume the following form:

(3) $${\hat{e}}_{1}=2\pi \frac{e_{2}\times e_{3}}{e_{1}\cdot \left(e_{2}\times e_{3}\right)}=2\pi e_{1}$$

(4) $${\hat{e}}_{2}=2\pi \frac{e_{3}\times e_{1}}{e_{2}\cdot \left(e_{3}\times e_{1}\right)}=2\pi e_{2}$$

where **e**_3_ is the unit vector normal to the plane of the problem (Figure 1).

If the Bloch wave vector is expressed in the reciprocal space as follows:

(5) $$k=k_{1}{\hat{e}}_{1}+k_{2}{\hat{e}}_{2}$$

Then, from Equation (2), the following equation is obtained, which provides a periodic boundary condition for the dynamic analysis of the unit cell:

(6) $$u\left(r\right)=u\left(r_{0}\right)\exp\left[2\pi \left(n_{1}k_{1}+n_{2}k_{2}\right)\right]$$

## 4. Finite Element Approximation

**Figure 2:**
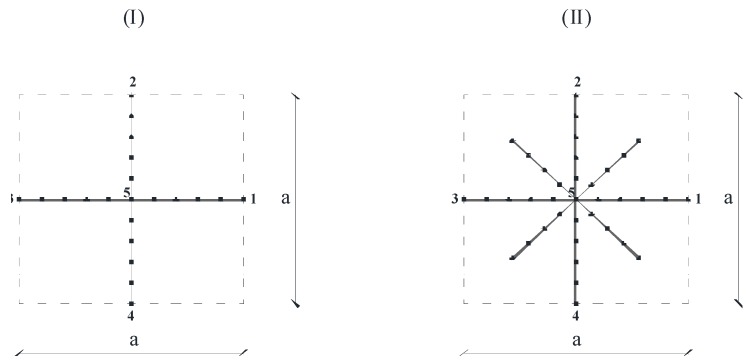
Examples of the FE mesh over the unit cell of a 2D square lattice material (a being the lattice constant).

The analysis of the free wave propagation in 2D lattice materials can be performed by modeling a unit cell only. Rigid connections are usually considered between the micro-beams of which the unit cell is composed. Examples of the finite element (FE) mesh over the unit cell are shown in Figure 2. In this figure, both a simple 2D square lattice materials (I) and a more elaborated one (II) are sketched, the last one being composed of auxiliary cantilever beams interconnected to the primary microstructure. It is worth noting that this second example is substantially the same as reported in [39].

**Figure 3:**
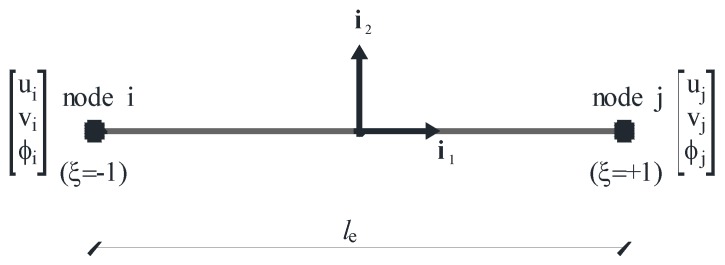
Finite element.

Regardless of how many micro-beams are interconnected to form the unit cell, the mesh here proposed is composed of finite elements characterized by six degrees of freedom (d.o.f.), including three d.o.f per each node p (p = node i or node j): the displacements ${\text{u}}_{\text{p}}$, ${\text{v}}_{\text{p}}$ along the directions $i_{1}$, $i_{2}$ and the rotation $\varphi_{\text{p}}$ about the axis $i_{3}$, where ($i_{1}$, $i_{2}$, $i_{3}$) are the local co-ordinates depicted in Figure 3, with $i_{1}$ aligned from node i to node j and $i_{3}$ = $e_{3}$. It is important to highlight that, without losing generality, the first four nodes of the mesh are supposed to be located at the connections with the adjacent cells, while the fifth node is located at the center of the unit cell under consideration (Figure 2).

Let $u_{\text{e}}$ be the numeric vector collecting all degrees of freedom related to a generic finite element (Figure 3):

(7) $$u_{\text{e}}={\left[{\text{u}}_{\text{i}},{\text{v}}_{\text{i}},\varphi_{\text{i}},{\text{u}}_{\text{j}},{\text{v}}_{\text{j}},\varphi_{\text{j}}\right]}^{\text{T}}$$

Accounting for a general orientation of the finite element, it is useful to express the numeric vector $u_{\text{e}}$, which refers to the local co-ordinates system ($i_{1}$, $i_{2}$, $i_{3}$) as a function of the nodal displacements referred, instead, to the global co-ordinates system ($e_{1}$, $e_{2}$, $e_{3}$ = $i_{3}$): $U_{\text{e}}={\left[{\text{U}}_{\text{i}},{\text{V}}_{\text{i}},\Phi_{\text{i}},{\text{U}}_{\text{j}},{\text{V}}_{\text{j}},\Phi_{\text{j}}\right]}^{\text{T}}$ with $\Phi_{\text{i}}$ = $\varphi_{\text{i}}$ and $\Phi_{\text{j}}$ = $\varphi_{\text{j}}$. It results:

(8) $$u_{\text{e}}=\left[\begin{array}{cccccc} \cos\alpha & sen\alpha & 0 & 0 & 0 & 0\\ -sen\alpha & \cos\alpha & 0 & 0 & 0 & 0\\ 0 & 0 & 1 & 0 & 0 & 0\\ 0 & 0 & 0 & \cos\alpha & sen\alpha & 0\\ 0 & 0 & 0 & -sen\alpha & \cos\alpha & 0\\ 0 & 0 & 0 & 0 & 0 & 1 \end{array}\right]U_{\text{e}}=QU_{\text{e}}$$

α being the angle from $e_{1}$ to $i_{1}$

By virtue of the next position:

(9) $$\left[\begin{array}{c} {\text{v}}_{\text{i}}^{\prime }\\ {\text{v}}_{\text{j}}^{\prime } \end{array}\right]=\frac{1}{1+\eta }\left[\begin{array}{cccccc} 0 & -\frac{\eta }{l_{\text{e}}} & \left(1+\frac{\eta }{2}\right) & 0 & \frac{\eta }{l_{\text{e}}} & -\frac{\eta }{2}\\ 0 & -\frac{\eta }{l_{\text{e}}} & -\frac{\eta }{2} & 0 & \frac{\eta }{l_{\text{e}}} & \left(1+\frac{\eta }{2}\right) \end{array}\right]\;\left[\begin{array}{c} {\text{u}}_{\text{i}}\\ {\text{v}}_{\text{i}}\\ \varphi_{\text{i}}\\ {\text{u}}_{\text{j}}\\ {\text{v}}_{\text{j}}\\ \varphi_{\text{j}} \end{array}\right]$$

where *η* indicates the following dimensionless quantity accounting for the influence of the shear deformations

(10) $$\eta =\frac{12EI}{GA_{S}l_{e}^{2}}$$

the displacements field ${\left[u,v,\varphi \right]}^{\text{T}}$, expressed in the local co-ordinates, can be interpolated over the generic finite element as follows:

(11) $$\left[\begin{array}{c} u\\ v\\ \varphi \end{array}\right]=\left[\begin{array}{cccccc} f_{1} & 0 & 0 & f_{2} & 0 & 0\\ 0 & \lambda_{{\text{v}}_{1}} & \lambda_{\varphi_{1}} & 0 & \lambda_{{\text{v}}_{2}} & \lambda_{\varphi_{2}}\\ 0 & 0 & f_{1} & 0 & 0 & f_{2} \end{array}\right]\;u_{\text{e}}=Nu_{\text{e}}$$

In Equation (10), the symbols *E*, *G*, *I* and $A_{\text{s}}$ indicate the Young modulus of the bulk material, the shear modulus, the inertia of the cross-section and the area of the cross-section incorporating the shear correction ($A_{\text{s}}={\text{k}}_{\text{s}}A$). With reference to the well-known plane strain hypothesis, both *I* and $A_{\text{s}}$ are evaluated per unit length along $i_{3}$. Furthermore, $l_{\text{e}}$ denotes the length of the finite element.

Moreover, in Equation (11), the symbols $f_{1}$ and $f_{2}$ denote the classical linear Lagrange polynomials, while the symbols $\lambda_{{\text{v}}_{1}}$, $\lambda_{\varphi_{1}}$, $\lambda_{{\text{v}}_{2}}$ and $\lambda_{\varphi_{2}}$ denote four enhanced shape functions, which allow the incorporation of the effect of shear deformations. It results:

(12) $$f_{1}=\frac{1}{2}\left(1-\xi \right)$$

(13) $$f_{2}=\frac{1}{2}\left(1+\xi \right)$$

(14) $$\lambda_{{\text{v}}_{1}}={\text{h}}_{\text{10}}-\frac{1}{l_{\text{e}}}\frac{\eta }{\left(1+\eta \right)}\left({\text{h}}_{\text{11}}+{\text{h}}_{21}\right)$$

(15) $$\lambda_{\varphi_{1}}=\frac{2+\eta }{2\left(1+\eta \right)}{\text{h}}_{\text{11}}-\frac{\eta }{2\left(1+\eta \right)}{\text{h}}_{\text{21}}$$

(16) $$\lambda_{{\text{v}}_{2}}={\text{h}}_{\text{20}}+\frac{1}{l_{\text{e}}}\frac{\eta }{\left(1+\eta \right)}\left({\text{h}}_{\text{11}}+{\text{h}}_{21}\right)$$

(17) $$\lambda_{\varphi_{2}}=-\frac{\eta }{2\left(1+\eta \right)}{\text{h}}_{\text{11}}+\frac{2+\eta }{2\left(1+\eta \right)}{\text{h}}_{\text{21}}$$

with ξ indicating the normalized axial coordinate (|ξ|≤1, Figure 3) and ${\text{h}}_{\text{pq}}$ being four appropriate interpolating cubic functions (p = 1,2), (q = 0,1):

(18) $${\text{h}}_{\text{10}}=\frac{1}{4}\left(2-3\xi +\xi^{3}\right)$$

(19) $${\text{h}}_{\text{11}}=\frac{l_{\text{e}}}{8}\left(1-\xi -\xi^{2}+\xi^{3}\right)$$

(20) $${\text{h}}_{\text{20}}=\frac{1}{4}\left(2+3\xi -\xi^{3}\right)$$

(21) $${\text{h}}_{\text{21}}=\frac{l_{\text{e}}}{8}\left(-1-\xi +\xi^{2}+\xi^{3}\right)$$

The finite element proposed is able to account for shear strains and is locking-free. These features, as it is well-known, are essential for short transient and wave propagation analysis. A Bernoulli-Euler element, in fact, exhibiting an infinite phase velocity, because the parabolic order of the equation of motion, is useless for simulating wave propagation.

According to the non-local beam model presented in [35] (see Appendix), the generalized stresses within the generic finite element can be expressed as a function of the nodal displacements:

(22) $$S={\left[N,\;M,\;Y,\;V\right]}^{\text{T}}=\mathrm{CB}u_{\text{e}}$$

where *N*, *M*, *Y* and *V* denote the generalized stresses of the beam model, **C** denotes the matrix given in Equation (A30) and **B** is a numeric matrix with the following entries:

(23) $$B=\left[\begin{array}{cccccc} f_{1}^{\prime } & 0 & 0 & f_{2}^{\prime } & 0 & 0\\ 0 & 0 & f_{1}^{\prime } & 0 & 0 & f_{2}^{\prime }\\ 0 & \frac{1}{2}\lambda_{{\text{v}}_{1}}^{\prime \prime } & \frac{1}{2}\left(\lambda_{\varphi_{1}}^{\prime \prime }+f_{1}^{\prime }\right) & 0 & \frac{1}{2}\lambda_{{\text{v}}_{2}}^{\prime \prime } & \frac{1}{2}\left(\lambda_{\varphi_{2}}^{\prime \prime }+f_{2}^{\prime }\right)\\ 0 & \lambda_{{\text{v}}_{1}}^{\prime } & \left(\lambda_{\varphi_{1}}^{\prime }-f_{1}\right) & 0 & \lambda_{{\text{v}}_{2}}^{\prime } & \left(\lambda_{\varphi_{2}}^{\prime }-f_{2}\right) \end{array}\right]$$

with reference to the global coordinates, the element stiffness matrix $K_{\text{e}}$ assumes the following form:

(24) $$K_{\text{e}}=\frac{l_{e}}{2}\int_{-1}^{+1}Q^{\text{T}}B^{\text{T}}\mathrm{CBQ}d\xi$$

On the other hand, the kinetic energy can be expressed as follows:

(25) $$T=\frac{1}{2}\left[\frac{l_{e}}{2}\int_{-1}^{+1}\left(\rho A{\dot{u}}^{2}+\rho A{\dot{v}}^{2}+\rho I{\dot{\varphi }}^{2}\right)d\xi \right]={\dot{u}}_{\text{e}}^{\text{T}}M_{\text{e}}{\dot{u}}_{\text{e}}$$

where the consistent mass matrix $M_{\text{e}}$ is computed over the generic finite element by means of:

(26) $$M_{\text{e}}=\frac{1}{2}\rho A\frac{l_{e}}{2}\int_{-1}^{+1}Q^{\text{T}}N^{\text{T}}\left[\begin{array}{ccc} 1 & 0 & 0\\ 0 & 1 & 0\\ 0 & 0 & r^{2} \end{array}\right]\mathrm{NQ}d\xi \;\text{with}\;r=\sqrt{I/A}$$

By standard procedures, the equations of motion can be assembled in the following matrix form:

(27) $$M_{\text{g}}{\ddot{U}}_{\text{g}}+K_{\text{g}}U_{\text{g}}=F$$

where $M_{\text{g}}$ and $K_{\text{g}}$ are the overall mass and stiffness matrices of the unit cell and $U_{\text{g}}={\left[U_{1},U_{2},U_{3},U_{4},U_{5},\ldots ,U_{N}\right]}^{\text{T}}$ and F denote the nodal displacements and external forces vectors in the global reference system (Ω, $e_{1}$, $e_{2}$), respectively, Nbeing the number of nodes over the unit cell. If a plane elastic wave with angular frequency ω is considered, Equation (27) assumes the new form:

(28) $$(-\omega^{2}M_{\text{g}}+K_{\text{g}})U_{\text{g}}=F$$

The last form of the equations of motion (Equation (28)) must be coupled with the periodic boundary conditions given by Equation (6):

(29) $$U_{1}=U_{3}\exp\left(2\pi k_{1}\right)$$

(30) $$U_{2}=U_{4}\exp\left(2\pi k_{2}\right)$$

where $U_{1}$, $U_{2}$, $U_{3}$ and $U_{4}$ are the nodal displacements relative to node 1 to 4, indicated in Figure 2 (connections with the four adjacent cells). 

By means of Equations (29) and (30), the number of degrees of freedom is equal to $N_{\text{dof}}=3\times \left(N-2\right)$. The global displacements vector $U_{\text{g}}={\left[U_{1},U_{2},U_{3},U_{4},U_{5},\ldots ,U_{N}\right]}^{\text{T}}$, in fact, can be expressed as a function of a reduced displacements vector $U_{\text{r}}={\left[U_{3},U_{4},U_{5},\ldots ,U_{N}\right]}^{\text{T}}$, according to the following relationship:

(31) $$U_{\text{g}}=HU_{\text{r}}$$

where the matrix H works as a transfer operator:

(32) $$H=\left[\begin{array}{cccccc} {\text{c}}_{1}I & 0 & 0 & 0 & \cdots & 0\\ 0 & {\text{c}}_{2}I & 0 & 0 & \cdots & 0\\ I & 0 & 0 & 0 & \cdots & 0\\ 0 & I & 0 & 0 & \cdots & 0\\ 0 & 0 & I & 0 & \cdots & 0\\ 0 & 0 & 0 & \ddots & \ddots & \vdots \\ \vdots & \vdots & \vdots & \ddots & I & 0\\ 0 & 0 & 0 & \cdots & 0 & I \end{array}\right],\;{\text{c}}_{1}=\exp\left(2\pi k_{1}\right),\;{\text{c}}_{2}=\exp\left(2\pi k_{2}\right)$$

with:

(33) $$I=\left[\begin{array}{ccc} 1 & 0 & 0\\ 0 & 1 & 0\\ 0 & 0 & 1 \end{array}\right]$$

(34) $$0=\left[\begin{array}{ccc} 0 & 0 & 0\\ 0 & 0 & 0\\ 0 & 0 & 0 \end{array}\right]$$

Finally, the equations of motion of the unit cell can be expressed in the following form:

(35) $$(-\omega^{2}H^{\text{T}}M_{\text{g}}H+H^{\text{T}}K_{\text{g}}H)U_{\text{r}}=H^{\text{T}}F$$

**Figure 4:**
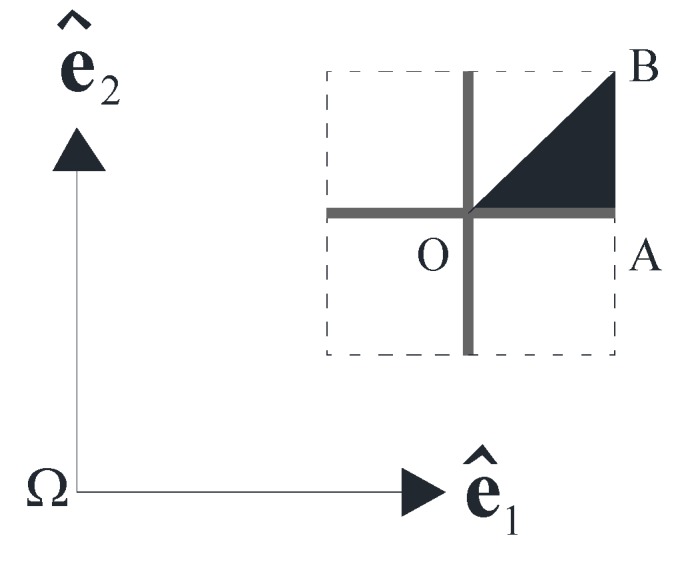
Irreducible part of the first *Brillouin* zone.

The condition F=0 (free wave motion) implies that the study of the wave transmission within the periodic square lattice material reduces to the eigenvalue problem of Equation (35). For fixed values of $k_{1}$ and $k_{2}$, to be expressed in the reciprocal space (${\hat{e}}_{1}$, ${\hat{e}}_{2}$) according to Equation (5), the frequencies of the wave propagation coincide with the eigenvalues of the problem formulated in Equation (35). Varying $k_{1}$ and $k_{2}$ along the boundary of the irreducible part of the first *Brillouin* zone, the band structure of the lattice material can be detected. In Figure 4, the filled region (triangle **OAB)** indicates the irreducible part of the first *Brillouin* zone concerning the square topology under consideration.

## 5. Numerical Results

In order to elucidate the investigation approach, the finite element model presented in the previous section has been applied for studying the dynamic behavior of a 2D square lattice material. More in detail, a parametric analysis has been carried out by varying the role of the microstructure length *l*. The example under consideration concerns the scheme (I) shown in Figure 2, the geometry and the mechanical parameters being fixed, as indicated in Table 1.

**Table 1:** Geometry and mechanical parameters.

**lattice constant:**	a=1.0 mm
cross-section area per unit length along i_3_	$A=1.000\times 10^{-1}{\text{ mm}}^{2}/\text{mm}$
cross-section shear area per unit length along i_3_	$A_{\text{s}}=8.333\times 10^{-2}{\text{ mm}}^{2}/\text{mm}$
flexural inertia per unit length along i_3_	$I=8.333\times 10^{-5}{\text{ mm}}^{4}/\text{mm}$
Young modulus	$E=2.100\times 10^{5}{\text{ N/mm}}^{2}$
shear modulus	$G=8.077\times 10^{4}{\text{ N/mm}}^{2}$
Poisson ratio	ν=0.3
mass density	$\rho =7\text{.850}\times {\text{10}}^{\text{-6}}{\text{ Kg/mm}}^{3}$

The mesh used for the numerical simulations has been obtained by dividing both the horizontal beam and the vertical one shown in Figure 2 (I) by 50 finite elements, each one. The overall number of finite element results equal to 100, while the overall number of nodes (*N*) is equal to 101. The final number of degrees of freedom (*N*_dof_) is thereby equal to 297. Many tests have been carried out in order to check the convergence rate and to assess the accuracy of the numerical solutions.

The following values of the dimensionless microstructure parameter λ=l/a have been considered in the analysis: {0.00, 0.01, 0.05, 0.10}. It is important to remark that the first value (λ=0.00) implies that the size-effect is not present.

The eigenvalue problem given by Equation (35) has been solved via a call to a dedicated routine belonging to the IMSL Math Library. Thus, the frequency values have been identified and represented in a dimensionless form $\tilde{\omega }$ by means of:

(36) $$\tilde{\omega }=\omega /\omega_{1}$$

where $\omega_{1}$ denotes the first bending resonance frequency of a pinned-pinned beam with the same properties given in Table 1, its length being equal to the lattice constant *a*:

(37) $$\omega_{1}=\frac{\pi^{2}}{a^{2}}\sqrt{\frac{EI}{\rho A}}$$

**Figure 5:**
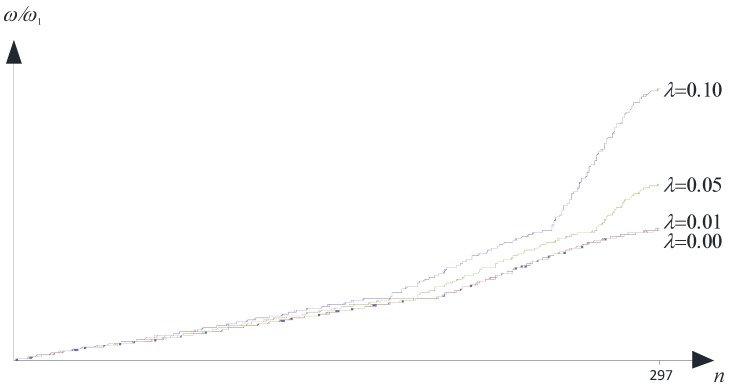
Dimensionless frequencies *versus* *n* (fixed point on the boundary OABO).

In Figure 5, a typical diagram of $\tilde{\omega }$ *versus* the modal number *n* is presented, with n=1, 2, ... , 297. The figure refers to fixed values of *k*_1_ and *k*_2_ (*i.e.*, a well-defined point on the boundary of the irreducible part of the first *Brillouin* zone).

As it is easy to argue, the size-effect is found to be strongly relevant within the high frequency region when dealing with *λ* > 0.01.

For what concerns the dynamic behavior within the low frequency region, Table 2 indicates also in this case a considerable influence of the parameter λ.

With respect to the corresponding predictions obtained by assuming *λ* = 0.00, relevant variations emerge, being the maximum percentage increase (+93%) found at point A when *λ* = 0.10.

**Table 2:** Ten lowest frequency values (dimensionless).

	*λ* = 0.00			*λ* = 0.01			*λ* = 0.05			*λ* = 0.10		
	O	A	B	O	A	B	O	A	B	O	A	B
1	0.00	0.15	1.79	0.00	0.15	1.80	0.00	0.19	1.85	0.00	0.29	1.89
2	0.00	1.83	1.81	0.00	1.83	1.81	0.00	1.88	1.88	0.00	1.91	1.94
3	4.21	4.04	4.35	4.27	4.09	4.40	5.40	5.18	5.41	7.17	6.91	7.06
4	4.21	4.50	4.35	4.27	4.56	4.41	5.40	5.62	5.43	7.17	7.34	7.11
5	6.07	6.17	6.27	6.16	6.26	6.37	7.88	7.99	8.12	10.74	10.87	11.02
6	8.60	8.70	8.80	8.70	8.81	8.91	10.53	10.65	10.75	12.94	13.06	13.17
7	15.62	15.32	15.33	15.77	15.46	15.48	18.10	17.76	17.77	20.66	20.30	20.27
8	15.62	15.64	15.34	15.77	15.78	15.48	18.10	18.10	17.77	20.66	20.63	20.27
9	20.49	20.60	20.72	20.72	20.83	20.95	24.62	24.71	24.82	29.29	29.31	29.31
10	22.97	23.08	23.18	23.18	23.28	23.38	26.45	26.54	26.61	30.14	30.12	30.06

## 6. Conclusions

As highlighted in a number of papers, the dynamic behavior of a square lattice material is significantly affected by the underlying microstructure. Due to the spatial periodicity, the hypothesis of infinite lattice points allows us to reduce the analysis to the unit cell and to investigate the dynamic behavior by means of the Bloch theorem.

It has been found by many authors that the natural frequencies predicted via a non-local beam model are always higher than those evaluated by classical beam models, due to the increased bending stiffness related to the so-called size-effect.

A more refined beam model is therefore required. 

For the scope of a reliable evaluation of the existence, position and width of frequency band gaps, in the opinion of the authors, the study of a 2D lattice materials should account for possible size-effects.

The paper provides a simple 1D finite element, developed in accordance with a simplified couple stress theory, which is able to simulate the size-effect via a unique microstructural parameter. Shear deformations are also accounted for.

The numerical results confirm the influence of the size-effect on the dynamic behavior of 2D periodic materials.

## Appendix

In accordance with higher order theories, the local equilibrium conditions require the presence of couple stresses m=Mn (moment per unit area) in addition to the classical Cauchy stresses t=Tn, while with respect to a global continuum, equilibrium equations can be expressed as follows:

(A1) $$\int_{V}\rho b\text{dv}+\int_{\partial V}t_{n}\text{ds}=0$$

(A2) $$\int_{V}\left[(x-x_{o})\wedge \rho b+\rho c\right]\text{dv}+\int_{\partial V}\left[(x-x_{o})\wedge t_{n}+c_{n}\right]\text{ds}=0$$

In Equations (A1) and (A2), *V* indicates an arbitrary volume of a deformable body bounded by ∂V, the symbol **n** denotes the outer normal to ∂V, **b** and **c** denote, respectively, the body force and the body couple per unit mass, ρ is the mass density, **x** − **xo** denotes the distance vector of a generic point from an arbitrary pole **O**. Furthermore, $t_{n}$ and $c_{n}$ denote, respectively, the traction and couple (per unit surface) acting on the boundary of the body.

The divergence theorem can be invoked in order to transform the 2D integrals in Equations (A1) and (A2):

(A3) $$\int_{V}(\rho b+\text{Div }T)\text{ dv}=0$$

(A4) $$\int_{V}\left[\text{Div }M+\rho c+2w_{\text{T}}\right]\text{dv}=0$$

In Equation (A4), the symbol $w_{\text{T}}$ represents the axial vector related to the skew part of the stress tensor **T**. Since the volume *V* is arbitrary, the volume dependence can be discarded, leading to the following equilibrium equations:

(A5) Div T+ρb=0 in V

(A6) $$\text{Div }M+\rho c+2w_{\text{T}}=0\;\text{in V}$$

(A7) $$\mathrm{Tn}=t_{n}\;\text{on}\;\partial V$$

(A8) $$\mathrm{Mn}=c_{n}\;\text{on}\;\partial V$$

Due to the presence of the couple stresses, Equation (A6) implies that the Cauchy stress tensor **T** is not symmetric. Then, it can be decomposed as follows:

(A9) T=S+L

(A10) $$S=\frac{1}{2}\left(T+T^{\text{T}}\right)$$

(A11) $$L=\frac{1}{2}\left(T-T^{\text{T}}\right)$$

Furthermore, it results:

(A12) $$w_{\text{T}}=-\frac{1}{2}{\text{e}}_{\text{ijk}}{\text{L}}_{\text{jk}}e_{\text{i}}$$

On the other hand, at the local scale, the deformation measures are represented by the following two tensors:

(A13) $$\Gamma_{\text{ij}}=\frac{\partial {\text{u}}_{\text{j}}}{\partial x_{\text{i}}}-{\text{e}}_{\text{ijk}}\theta_{\text{k}}$$

(A14) $${Κ}_{\text{ij}}=\frac{\partial \theta_{\text{j}}}{\partial x_{\text{i}}}$$

The symbols Γ and Κ denote the (small) strain and curvature tensor, respectively. It is easy to verify that the following relationships exist:

(A15) $${\text{E}}_{\text{ij}}=\frac{1}{2}\left(\Gamma_{\text{ij}}+\Gamma_{\text{ji}}\right)=\frac{1}{2}\left[\frac{\partial {\text{u}}_{\text{i}}}{x_{\text{j}}}+\frac{\partial {\text{u}}_{\text{j}}}{x_{\text{i}}}\right]$$

(A16) $${\text{A}}_{\text{ij}}=\frac{1}{2}\left(\Gamma_{\text{ij}}-\Gamma_{\text{ji}}\right)={\text{e}}_{\text{ijk}}\left(\omega_{\text{k}}-\theta_{\text{k}}\right)$$

where ${\text{u}}_{\text{i}}$ and $\theta_{\text{i}}$ denote the generic components of the displacement field and the micro-rotation field (i = 1,2,3). The symmetric part of Γ in Equation (A15) denotes the classical infinitesimal strain tensor, while the skew part in Equation (A16) accounts for the difference between the macro rotations $\omega_{\text{k}}\text{=}\frac{1}{2}{\text{e}}_{\text{ijk}}\frac{\partial {\text{u}}_{\text{j}}}{x_{\text{i}}}$ and the micro rotations $\theta_{\text{k}}$ (k = 1,2,3). As a consequence, the virtual work done by the internal stresses assumes the following final expression:

(A17) $$\delta L_{i}=\int_{V}\left(S\cdot \delta E+L\cdot \delta A+M\cdot \delta K\right)\text{dv}$$

It is worth noting that if no difference between the micro-rotations and macro-rotations occurs, the strain tensor **A** vanishes and the continuum model reduces to couple stress theory. In this case, the anti-symmetric tensor **L** does not contribute to the work done by the internal stresses. More precisely, the derivation of the couple stress theory [29] from the general Cosserat theory [25] has been investigated in [32], where a dimensionless number drives the transition. Unfortunately, the definition of both the Young modulus and the shear modulus fall in defect if the couple stress theory is interpreted as a special case of the micro-polar (Cosserat) theory.

Whereas, the link from the classical couple stress theory to the simplified form proposed in [35] is founded upon the following additional equilibrium equation for the moment of couples:

(A18) $$\int_{V}\left[(x-x_{o})\wedge \left(\rho c+2w_{T}\right)\right]\text{dv}+\int_{\partial V}\left[(x-x_{o})\wedge c_{n}\right]\text{ds}=0$$

that, by virtue of the divergence theorem, becomes:

(A19) $$\int_{V}\left[Z\left(\text{Div }M+\rho c+2w_{\text{T}}\right)+2w_{\text{M}}\right]\text{dv}=0$$

In Equation (A19), **Z** indicates the skew tensor related to the distance vector (**x** – **x**_0_) and $w_{\text{M}}$ denotes the axial vector corresponding to the anti-symmetric part of **M**. By considering Equation (A6) into Equation (A19), it descends that the couple stress tensor **M** is symmetric.

The work done by the internal stresses assumes the following simplified form:

(A20) $$\delta L_{i}=\int_{V}\left(S\cdot \delta E+\bar{m}\cdot \delta \kappa \right)\text{dv}$$

where $\bar{m}$ indicates the deviatoric part of the couple stress tensor **M** and κ the symmetric part of the curvature tensor K.

(A21) $$\kappa =\frac{1}{2}\left[\nabla \theta +{\left(\nabla \theta \right)}^{\text{T}}\right]\;\text{with}\;\nabla \theta =\nabla \omega$$

(A22) $$\bar{m}=M-\text{a }I\;\text{with}\;\text{a }=\frac{1}{3}tr(M)$$

Finally, the mathematical expression of the deformation energy density w has been assumed as follows:

(A23) $$\text{w}=\frac{1}{2}\lambda {\left(trE\right)}^{2}+\mu \left(E\cdot E+l^{2}\kappa \cdot \kappa \right)$$

where *λ* and μ denote the Lame’s constants, while *l* is a microstructure length scale parameter.

In detail, with reference to the beam model proposed for the analysis of the unit cell, the displacement field is:

(A24) $${\text{u}}_{1}=u(x)-\varphi (x)y$$

(A25) $${\text{u}}_{2}=v(x)$$

(A26) $${\text{u}}_{3}=0$$

where ${\text{u}}_{1}$, ${\text{u}}_{2}$ and ${\text{u}}_{3}$ are the displacement field components along the local axes ($i_{1}$, $i_{2}$, $i_{3}$); *x* and *y* denote the rectangular coordinates referred to $i_{1}$ and $i_{2}$, respectively, and u(x), v(x) and φ(x) are the generalized displacements of the cross-section. It results:

(A27) $$E=\left[\begin{array}{ccc} \left(\frac{\partial u}{\partial x}-y\frac{\partial \varphi }{\partial x}\right) & \frac{1}{2}\left(\frac{\partial v}{\partial x}-\varphi \right) & 0\\ \frac{1}{2}\left(\frac{\partial v}{\partial x}-\varphi \right) & 0 & 0\\ 0 & 0 & 0 \end{array}\right]$$

(A28) $$\kappa =\left[\begin{array}{ccc} 0 & 0 & \frac{1}{4}\left(\frac{\partial^{2}v}{\partial x^{2}}+\frac{\partial \varphi }{\partial x}\right)\\ 0 & 0 & 0\\ \frac{1}{4}\left(\frac{\partial^{2}v}{\partial x^{2}}+\frac{\partial \varphi }{\partial x}\right) & 0 & 0 \end{array}\right]$$

The constitutive equations assume thereby the following form:

(A29) $${\left[N,\;M,\;Y,\;V\right]}^{\text{T}}=C{\left[\frac{\partial u}{\partial x},\;\frac{\partial \varphi }{\partial x},\;\frac{1}{2}\left(\frac{\partial \varphi }{\partial x}+\frac{\partial^{2}v}{\partial x^{2}}\right),\;\left(-\varphi +\frac{\partial v}{\partial x}\right)\right]}^{\text{T}}$$

where **C** denotes the local stiffness matrix:

(A30) $$C=\left[\begin{array}{cccc} \frac{E\left(1-\nu \right)A}{\left(1+\nu \right)\left(1-2\nu \right)} & 0 & 0 & 0\\ 0 & \frac{E\left(1-\nu \right)I}{\left(1+\nu \right)\left(1-2\nu \right)} & 0 & 0\\ 0 & 0 & GAl^{2} & 0\\ 0 & 0 & 0 & GA_{\text{s}} \end{array}\right]$$

In Equation (A29), * N*, *M*, *Y* and *V* denote the generalized stresses of the beam model (axial force, bending moment, additional bending moment due to the size-effect, shear force), while $\frac{\partial u}{\partial x}$, $\frac{\partial \varphi }{\partial x}$, $\frac{1}{2}\left(\frac{\partial \varphi }{\partial x}+\frac{\partial^{2}v}{\partial x^{2}}\right)$ and $\left(-\varphi +\frac{\partial v}{\partial x}\right)$ are the conjugated strains. Finally, symbols *E*, *G* and ν denote the Young modulus, the shear modulus and the Poisson coefficient, which are related to the Lame’s constants:

(A31) $$\lambda =\frac{E\nu }{\left(1+\nu \right)\left(1-2\nu \right)}$$

(A32) $$\mu =\frac{E}{2\left(1+\nu \right)}$$

In relation to the characteristic length *l*, a mechanical definition has been given in [27].
